# High risk of developing subsequent epilepsy in patients with sleep-disordered breathing

**DOI:** 10.1371/journal.pone.0173491

**Published:** 2017-03-14

**Authors:** Tomor Harnod, Yu-Chiao Wang, Cheng-Li Lin, Chun-Hung Tseng

**Affiliations:** 1 Department of Neurosurgery, Hualien Tzu Chi General Hospital, Buddhist Tzu Chi Medical Foundation, Hualien, Taiwan; 2 College of Medicine, Tzu Chi University, Hualien, Taiwan; 3 Management Office for Health Data, China Medical University Hospital, Taichung, Taiwan; 4 College of Medicine, China Medical University, Taichung, Taiwan; 5 Graduate Institute of Clinical Medical Science and School of Medicine, College of Medicine, China Medical University, Taichung, Taiwan; 6 Department of Neurology, China Medical University Hospital, Taichung, Taiwan; Istituto Di Ricerche Farmacologiche Mario Negri, ITALY

## Abstract

**Purpose:**

Sleep-disordered breathing (SDB) is often associated with other medical disorders. Whether SDB interacts with other factors for developing subsequent epilepsy remains unclear.

**Methods:**

This population-based cohort study was conducted using the National Health Insurance Research Database of Taiwan. Patients aged >20 years and diagnosed with SDB between 2000 and 2010 comprised the SDB cohort (n = 138,507), and their data were compared with those of the comparison cohort (n = 138,507). The adjusted hazard ratio (aHR) for epilepsy was calculated using a multivariate Cox proportional hazards model.

**Results:**

The SDB cohort had an increased risk of epilepsy (aHR = 1.50, 95% confidence interval [CI] = 1.36–1.66). The sex-stratified analysis revealed a significant adjusted hazard ratio (aHR) for epilepsy with a 1.51-fold higher risk for female patients, and also a significantly 1.49-fold higher risk for male patients in the SDB cohort. Although epilepsy incidence increased with age in both cohorts, different age groups in the SDB cohort all had a significantly higher risk of developing epilepsy than comparison cohort.

**Conclusion:**

This population-based cohort study indicates that patients with SDB are at a high risk of developing subsequent epilepsy, in both sexes and all age groups.

## Introduction

Circadian rhythm regulates sleep, which is vital for health. Sleep disorders (SLDs) are common health concerns in the general population that can affect the quality of daily life of patients. SLDs with presentation of insomnia have been associated with other neurological and psychological diseases and may profoundly affect the physiology, behavior, and daily abilities of patients during waking hours [1]. For example, Vgontzas et al [2] reported that insomnia increased the mortality risk for men by 4-fold after adjustment for confounders, whereas the mortality risk for females did not increase. Compared with SLD, epilepsy has a lower prevalence of 0.5%–1.0% in general populations worldwide [3,4]. People with epilepsy have a high risk of premature death without definite sex difference, mostly from severe vascular diseases [5,6], and a 2.5- to 3.21-fold mortality risk has been reported in patients with epilepsy compared with those without epilepsy [7,8].

Epilepsy develops because of imbalanced expression of the excitatory and inhibitory ion channels in the brain. The electrophysiological instability in the cortex registers as a spike in an electroencephalogram. Occasionally, spikes with hypersynchronization lead to an epileptic seizure. Sleep deprivation increases seizure susceptibility and often provokes epileptic seizures [9,10]. Among SLDs, sleep apnea, also known as sleep-disordered breathing (SDB), is thought as one of severe conditions and often associated with other medical disorders, which probably including epilepsy. A high prevalence of SDB [11] and its significant association with epilepsy [12] have been reported in older populations. However, although the association between SLDs and epilepsy has been studied a lot, the causality or relationship between general patients with SDB and epilepsy development still remains unclear. Whether SDB is a single predisposing factor or interacts with other factors to develop epilepsy (thus substantially affecting the quality of life in and lifespan of patients with SDB) requires more evidence for optimizing the daily practice of patient care. Therefore, in this population-based cohort study, we enrolled patients with SDB from Taiwan’s National Health Insurance Research Database (NHIRD) and analyzed their data according to various factors.

## Methods and materials

### Data source

Taiwan launched the National Health Insurance (NHI) program in 1995, which is operated by a single-buyer, the government of Taiwan. Medical reimbursement specialists and peer reviewers scrutinize all insurance claims. The present prospective population-based cohort study was conducted using the Longitudinal Health Insurance Database (LHID), a subset of the NHIRD, established by the National Health Research Institutes, Department of Health, Taiwan. The NHIRD contains data, such as registration files and claims data, from the NHI program that are used for reimbursements for approximately 99% of Taiwan’s population. Data in the LHID, which was established for facilitating research and includes the 1996–2011 claims data of one million people covered by the NHI program, are derived from files in the NHIRD. There are no significant differences in the distributions of sex and age between the LHID and all beneficiaries of the NHI.

### Data availability statement

All data and related metadata were deposited in an appropriate public repository in the National Health Research Institutes (NHRI). The data on the study population that were obtained from the NHIRD (http://nhird.nhri.org.tw/en/index.html) are maintained in the NHIRD (http://nhird.nhri.org.tw/). The NHRI is a nonprofit foundation established by the government. Only citizens of the Republic of China (Taiwan) who fulfill the requirements of conducting research projects are eligible to apply for the NHIRD. The use of NHIRD is limited to research purposes only. Applicants must follow the Computer-Processed Personal Data Protection Law (http://www.winklerpartners.com/?p=987) and related regulations of National Health Insurance Administration and NHRI, and an agreement must be signed by the applicant and his/her supervisor upon application submission. All applications are reviewed for approval of data release.

### Ethics statement

The NHIRD encrypts patient personal information to protect privacy and provides researchers with anonymous identification numbers associated with relevant claims information, including sex, date of birth, medical services received, and prescriptions. Therefore, patient consent is not required to access the NHIRD. This study was approved to fulfill the condition for exemption by the Institutional Review Board (IRB) of China Medical University (CMUH104-REC2-115-CR1). The IRB also specifically waived the consent requirement.

### Study population

We conducted a prospective population-based cohort study to analyze the association between SDB and epilepsy. Our definitions of specific disease diagnoses were based on the International Classification of Diseases, Ninth Revision, Clinical Modification (ICD-9-CM). The SDB cohort included patients newly diagnosed with SDB (ICD-9-CM 780.5) between 2000 and 2010 (n = 138,507). The date of SDB diagnosis was defined as the index date. The comparison cohort was formed by randomly selecting control participants, matched for age (5-year intervals), sex, and the index year (n = 138,507), for each patient with SDB. None of the controls had a history of SLD (ICD-9-CM 307.4 and 780.5). The dates of randomly selected outpatient or inpatient visits during the index years were used as the index dates for the controls. All the participants were older than 20 years. Patients diagnosed with epilepsy (ICD-9-CM 345) before the index date and patients with missing data (sex and age) were excluded. The follow-up duration was calculated from the index date until withdrawal from the NHI, occurrence of epilepsy, or December 31, 2011. Diagnoses of SDB and epilepsy were identified using the relevant ICD-9-CM codes.

Adulthood epilepsy may be idiopathic or may present as symptomatic epilepsy following a brain insult. The three major causes of symptomatic epilepsy are head injury (ICD-9-CM 850–854 and 959.01), stroke (ICD-9-CM 430–438), and brain tumors (ICD-9-CM 225, 191, 192, 194.3, and 194.4) [13]. Furthermore, potential risk factors for epilepsy, such as hypertension (ICD-9-CM 401–405), hyperlipidemia (ICD-9-CM 272), diabetes (ICD-9-CM 250), depression (ICD-9-CM 296.2, 296.3, 296.82, 300.4, 309.0, 309.1, 309.28, and 311), anxiety (ICD-9-CM 300.0, 300.2, 300.3, 308.3, and 309.81), and migraine (ICD-9-CM 346) were also identified at the baseline. Because sleep medications are often used worldwide for SLDs, the use of zolpidem and benzodiazepines (BZDs) in participants before the index date was recorded for further analysis. These risk factors were considered in this study for adjustment.

The accuracy of the coding in the NHIRD is reviewed by specialists on the basis of standard clinical criteria. When an instance of incorrect coding or misdiagnosis is identified, the associated hospital and physician are penalized heavily. Therefore, the diagnoses and codes for SDB, epilepsy, and comorbidities used in this study can be considered accurate and reliable.

### Statistical analysis

All statistical analyses were performed using SAS 9.4 software (SAS Institute, Cary, NC, USA), and incidence curves were plotted using R software (R Foundation for Statistical Computing, Vienna, Austria). The mean and standard deviation (SD) for the continuous variable (i.e., age) were calculated, and the differences in the SDB and comparison cohorts were analyzed using the Student *t* test.

We used the Mann–Whitney test to estimate the difference of median follow-up in both cohorts. The number and percentage of the categorical variables (i.e., sex and comorbidities) in both cohorts were calculated, and the differences were analyzed using the chi-squared test. We used the Cox proportional hazards model to calculate the crude hazard ratio (HR) and 95% confidence interval (CI) of epilepsy in both cohorts. The multivariable Cox proportional hazards model was employed to calculate the adjusted HR (aHR) and 95% CI of epilepsy in both cohorts after controlling for sex, age, hypertension, hyperlipidemia, diabetes, stroke, anxiety, depression, head injury, brain tumors, migraine, and zolpidem and BZD use. The interaction effect between SDB and other risk factors (including sex, age, hypertension, hyperlipidemia, diabetes, stroke, anxiety, depression, head injury, brain tumors, migraine, and sleep medication use) were estimated using the Cox proportional hazards model. Because the P value of the proportional hazards assumption was 0.01, we further analyzed the association between SDB and epilepsy, stratified by the follow-up duration, as the sensitivity analysis. Two-sided *p* < 0.05 was considered significant.

## Results

A total of 277,014 participants, 138,507 patients with SDB and 138,507 controls, were selected for the SDB and comparison cohorts (Table 1). The mean of age was similar in the SDB and comparison cohorts according to a Student *t* test. The distribution of sex and age was similar in the SDB and comparison cohorts (chi-squared test *p* = 0.99). The participants in both cohorts were predominantly female (61.6%) and aged <65 years (82.3%). The prevalence of comorbidities, such as hypertension, hyperlipidemia, diabetes, stroke, anxiety, depression, head injury, brain tumors, and migraine was higher in the SDB cohort than in the comparison cohort (*p* < 0.0001). The percentages of sleep medication use in the participants were 93.6% and 71.2% in the SDB and comparison cohorts, respectively. The median follow-up duration was more than 7 years in both cohorts (Mann–Whitney test *p* < 0.0001, Table 1).

**Table 1:** Demographics, history of comorbidity, and sleep medication use in the SDB and comparison cohorts.

	SDB				
	No (N = 138507)		Yes (N = 138507)		
	n	%	n	%	*p*-value
**Sex**^+^					0.99
Female	85374	61.6	85374	61.6	
Male	53133	38.4	53133	38.4	
**Age, year**^+^					0.99
20-45	64254	46.4	64254	46.4	
45-65	49759	35.9	49759	35.9	
≥65	24494	17.7	24494	17.7	
Mean (SD)^#^	48.0(16.2)		48.0(16.2)		0.31
**Comorbidity**^+^					
Hypertension	43054	31.1	56418	40.7	<.0001
Hyperlipidemia	29082	21.0	41917	30.3	<.0001
Diabetes	21591	15.6	27416	19.8	<.0001
Stroke	14543	10.5	21746	15.7	<.0001
Anxiety	9665	6.98	39842	28.8	<.0001
Depression	4029	2.91	21217	15.3	<.0001
Head injury	7998	5.77	11443	8.26	<.0001
Brain tumors	471	0.34	778	0.56	<.0001
Migraine	2858	2.06	9437	6.81	<.0001
**Sleep medication use**^+^					<.0001
Non	39907	28.8	8910	6.43	
Zolpidem	1036	0.75	1792	1.29	
BZD	82924	59.9	55762	40.3	
Both	14640	10.6	72043	52.0	
Median follow-up (IQR)*	7.31(5.11-9.36)		7.41(5.24-9.44)		<.0001

SDB, sleep-disordered breathing; SD, standard deviation; IQR, interquartile range

^+^Chi-square test

^#^Student’s t-test

*Mann-Whitney test.

**Fig 1:**
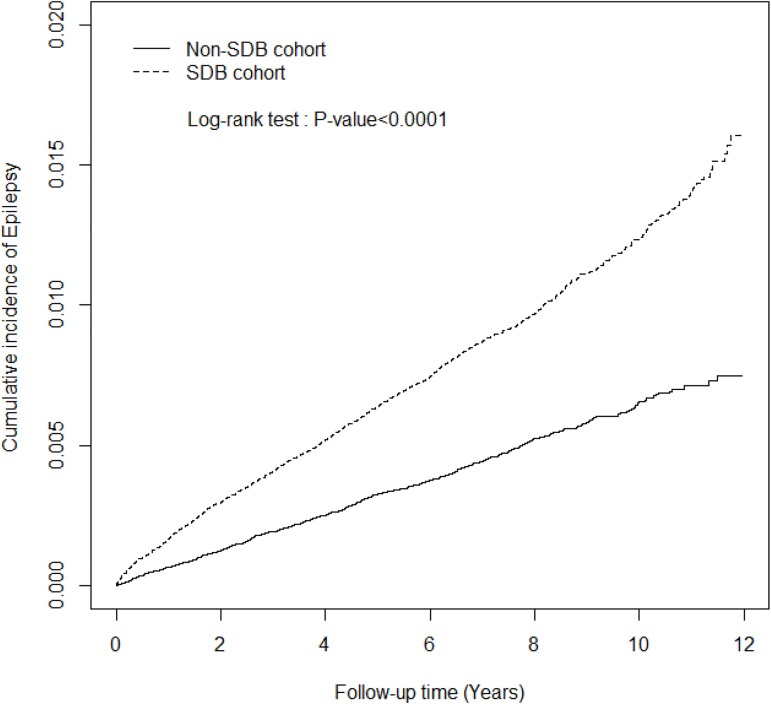
Cumulative incidence of epilepsy in the sleep-disordered breathing (SDB) (dashed line) and the comparison (solid line) cohorts.

During the follow-up period, the cumulative incidence of epilepsy was higher in the SDB cohort than in the comparison cohort (log-rank test *p* < 0.0001; Fig 1). In the SDB and comparison cohorts, 1277 and 640 occurrences of epilepsy were respectively noted (Table 2). The crude hazard ratio (HR) of epilepsy revealed a 1.97-fold risk in the SDB cohort compared with the comparison cohort (95% confidence interval [CI] = 1.79–2.16). After adjustment for age, sex, each comorbidity, and sleep medication use, the risk of epilepsy was 1.50-fold higher in the SDB cohort than in the comparison cohort (95% CI = 1.36–1.66). The sex-stratified analysis revealed a significant aHR for epilepsy with a 1.51-fold higher risk in female patients, and also a significantly 1.49-fold higher risk in male patients among the SDB cohort compared with those in the comparison cohort. Epilepsy incidence increased with age in both cohorts; however, different age groups in the SDB cohort all had a significantly higher risk of developing epilepsy than comparison cohort. Compared with the controls, the epilepsy risk increased by 1.43-fold in patients with SDB and one or more comorbidities (95% CI = 1.28–1.60), and 1.97-fold in patients with SDB and no comorbidities (95% CI = 1.47–2.65). Similarly, the epilepsy risk was significantly higher in patients with SDB and also with sleep medication use (zolpidem, BZD, or both) compared with that of the controls with sleep medications (aHR = 1.54 and 95% CI = 1.38–1.71) (Table 2).

**Table 2:** Incidence and adjusted hazard ratios of epilepsy stratified by sex, age, comorbidity (yes/no), and sleep medication use (yes/no) between the SDB and non-SDB cohorts.

	SDB							
		No			Yes		Relative to non-SDB cohort	
Variables	Event	PY	Rate	Event	PY	Rate	Crude HR (95% CI)	Adjusted HR^##^ (95% CI)
**Overall**	640	993419	0.64	1277	1008213	1.27	1.97(1.79, 2.16)***	1.50(1.36, 1.66)***
**Sex**								
Female	315	625076	0.50	626	637577	0.98	1.95(1.70, 2.23)***	1.51(1.31, 1.75)***
Male	325	368343	0.88	651	370636	1.76	1.99(1.74, 2.27)***	1.49(1.29, 1.72)***
**Age, year**								
20-45	122	467199	0.26	360	474552	0.76	2.91(2.37, 3.57)***	1.70(1.35, 2.12)***
45-65	227	370040	0.61	411	371746	1.11	1.80(1.53, 2.12)***	1.33(1.11, 1.58)**
≥65	291	156180	1.86	506	161916	3.13	1.68(1.45, 1.94)***	1.56(1.33, 1.82)***
**Comorbidity**^#^								
No	89	526191	0.17	106	300063	0.35	2.08(1.57, 2.75)***	1.97(1.47, 2.65)***
Yes^#^	551	467228	1.18	1171	708150	1.65	1.40(1.27, 1.55)***	1.43(1.28, 1.60)***
**Sleep medication use**^†^								
No	91	280057	0.32	15	58242	0.26	0.79(0.46, 1.36)	1.17(0.66, 2.05)
Yes^†^	549	713363	0.77	1262	949971	1.33	1.73(1.56, 1.91)***	1.54(1.38, 1.71)***

SDB, sleep-disordered breathing; PY, person-year; Rate, incidence rate (per 1,000 person-years); HR, hazard ratio

^##^Adjusted HR: multiple analysis including age, sex, each comorbidity and sleep medication use

^#^with one or more comorbidity of head injury, stroke, brain tumors, hypertension, hyperlipidemia, diabetes, depression, anxiety, and migraine

^†^with one or more sleep medication use

*p*-value for interaction between SDB and sex = 0.82

*p*-value for interaction between SDB and age groups <0.0001

*p*-value for interaction between SDB and comorbidity (yes/no) = 0.01

*p*-value for interaction between SDB and sleep medication use (yes/no) = 0.01

**p < 0.01

***p < 0.001.

The further analysis revealed that the *p*-value for interaction between SDB and sex was 0.82, the *p*-value for interaction between SDB and age groups was <0.0001, the *p*-value for interaction between SDB and comorbidity (yes/no) was 0.01, and the *p*-value for interaction between SDB and sleep medication use (yes/no) was 0.01 (Table 2).

Table 3 shows that the risk of epilepsy in the SDB cohort compared with that of the controls might decline over time, although the higher risk of developing epilepsy is persistently noted in patients with longer follow-up. The aHR declined from 2.44 (95% CI: 1.89–3.16) in participants with a follow-up of less than 1-year to 1.36 (95% CI = 1.14–1.62) in whom with 5-year follow-up or more.

**Table 3:** Incidence of epilepsy between the SDB and non-SDB cohorts stratified by follow-up duration.

	SDB							
		No			Yes		Compared to non-SDB cohort	
Variables	Event	PY	Rate	Event	PY	Rate	Crude HR (95% CI)	Adjusted HR^†^ (95% CI)
Follow-up time (year)								
<1	90	137342	0.66	231	137546	1.68	2.56(2.01, 3.27)***	2.44(1.89, 3.16)***
1–3	173	268067	0.65	326	269586	1.21	1.87(1.56, 2.25)***	1.47(1.20, 1.79)***
3–5	156	236748	0.66	275	240063	1.15	1.74(1.43, 2.12)***	1.24(1.01, 1.54)*
≥5	221	351263	0.63	445	361019	1.23	1.96(1.67, 2.30)***	1.36(1.14, 1.62)***

SDB, sleep-disordered breathing; PY, person-year; Rate, incidence rate (per 1,000 person-years); HR, hazard ratio

^**†**^Adjusted HR

multiple analysis including age, sex, each comorbidity and sleep medication use

*p < 0.05

***p < 0.001.

## Discussion

In this study, we demonstrated that the risk of epilepsy development persistently increased in the SDB cohort over time. Moreover, either in male or female sex, and whatever the patients with young, middle or old ages, they all are at a high risk of developing subsequent epilepsy. In pathogenesis, insomnia can increase the occurrence of interictal spikes and epileptic seizures even without evidence of apnea [14]. SDB or sleep apnea should theoretically affect human health markedly more than other SLDs without hypoxic effects do, and then develop subsequent epilepsy.

SDB may present with other diseases of the central nervous or respiratory system due to the hypoxic effects. We were surprised to find that there were only limited studies to reveal the association between SDB and developing epileptogenesis in the brain [12, 15]. SDB with hypoxia might increase the blood–brain barrier permeability, leading to brain edema, neurovascular uncoupling, and neuronal dysfunction and damage, those possibly result a higher risk of developing epilepsy [16]. Examining these data and the results of this study provided us with a new perspective on the pathogenesis link between these two types of brain disease, and a warning to clinicians about the higher possibility of epileptic seizures in patients with SDB.

Evidence has shown a link between higher epilepsy incidence and old age in the general population [4,12,13]. A sex-difference result was usually found in studies of epilepsy development [4,13]. In one of our previous studies with NHIRD data, it showed that female than male migraineurs had a higher risk of developing epilepsy [17]. It might be due to the frequency and severity of epileptic seizures may change during puberty, during the menstrual cycle, during pregnancy, and around the menopause stage in females [18–20]. On the other side, another previous study revealed a different sex-related result in a study about patients with insomnia, that showed increased mortal comorbidities in men but it did not noted in women [2]. Interestingly, the results of this study revealed that a higher risk for developing epilepsy was both noted in female and male patients with SDB, and the higher risk for epilepsy was demonstrated in all age groups. Therefore, we would hypothesize that although the combined effects are noted between SDB and sex, age, comorbidity, and sleep medication use for developing epilepsy, the insomnia with hypoxic apnea may vigorously affect the brain stability and thus increase the epilepsy risk. The SDB could be thought as a more powerful predisposing factor for developing subsequent epilepsy than sex, age, comorbidity, and sleep medication does.

### Study limitations

The strengths of this study are its nationwide population-based design and the representativeness of the study and comparison cohorts. However, this study has the following limitations. First, we could not approach the patients directly to confirm their sociodemographic or medical details because the research data are deidentified. Data on the epilepsy type, frequency, presence of aura, and medication are unavailable in the NHIRD; thus, a subgroup analysis could not be performed. Participants’ smoking habits, alcohol consumption, body mass index, weight, socioeconomic status, and family history, all of which may be confounding factors for epilepsy, are unavailable in the NHIRD. Second, evidence derived from a cohort study after adjustment for confounders may have several biases. Although our study design included adequate control for confounding factors, unmeasured or unknown confounders may have generated a bias. Third, the accuracy and value of the International Classification of Diseases, Ninth Revision, Clinical Modification (ICD-9-CM) coding and analyzed results in NHIRD studies have often been challenged. For rare events among large participant samples, the analysis results would be dubious because the statistical power is probably too low for detecting an interaction effect in the population. However, our statistical results show that the sample size was sufficient for achieving a significant interpretation of the phenomena observed in this study. Furthermore, several studies have confirmed the reliability of ICD-9-CM–based diagnoses and the valuable results of NHIRD designed studies [17,21,22]. Hence, the present study derived new evidence concerning the association between SBD and epilepsy and the predisposing effect for epilepsy development by SDB.

## Conclusions

The results of this population-based cohort study indicate an increased risk of developing subsequent epilepsy in patients with sleep apnea or SDB. The SDB could be thought as a more powerful predisposing factor for developing subsequent epilepsy than sex, age, comorbidity, and sleep medication use. Additional large, unbiased, population-based studies with finer data categorization and analyses are necessary to confirm our findings.

## Supporting information

S1 Checklist STROBE Checklist of items that should be included in reports of observational studies. (DOC)

## Abbreviations

SDB: Sleep-disordered breathing
aHR: adjusted hazard ratio
CI: confidence interval
SLDs: Sleep disorders
NHIRD: National Health Insurance Research Database
ICD-9-CM: International Classification of Diseases, Ninth Revision, Clinical Modification
LHID 2000: Longitudinal Health Insurance Database 2000
